# Graded changes in local functional connectivity of the cerebral cortex in young people with depression

**DOI:** 10.1017/S0033291725000510

**Published:** 2025-03-17

**Authors:** Alec J. Jamieson, Christopher G. Davey, Jesus Pujol, Laura Blanco-Hinojo, Ben J. Harrison

**Affiliations:** 1 Department of Psychiatry, The University of Melbourne, Parkville, Victoria, Australia; 2 MRI Research Unit, Department of Radiology, Hospital del Mar, Barcelona, Spain

**Keywords:** Major depressive disorder, local functional connectivity, resting-state, adolescent, young adult

## Abstract

**Background:**

Major depressive disorder (MDD) is marked by significant changes to the local synchrony of spontaneous neural activity across various brain regions. However, many methods for assessing this local connectivity use fixed or arbitrary neighborhood sizes, resulting in a decreased capacity to capture smooth changes to the spatial gradient of local correlations. A newly developed method sensitive to classical anatomo-functional boundaries, Iso-Distant Average Correlation (IDAC), was therefore used to examine depression associated alterations to the local functional connectivity of the brain.

**Method:**

One-hundred and forty-seven adolescents and young adults with MDD and 94 healthy controls underwent a resting-state functional magnetic resonance imaging (fMRI) scan. Whole-brain functional connectivity maps of intracortical neural activity within iso-distant local areas (5–10, 15–20, and 25–30 mm) were generated to characterize local fMRI signal similarities.

**Results:**

Across all spatial distances, MDD participants demonstrated greater local functional connectivity of the bilateral posterior hippocampus, retrosplenial cortex, dorsal insula, fusiform gyrus, and supplementary motor area. Local connectivity alterations in short and medium distances (5–10 and 15–20 mm) in the mid insula cortex were additionally associated with expressive suppression use, independent of depressive symptom severity.

**Conclusions:**

Our study identified increased synchrony of the neural activity in several regions commonly implicated in the neurobiology of depression. These effects were relatively consistent across the three distances examined. Longitudinal investigation of this altered local connectivity will clarify whether these differences are also found in other age groups and if this relationship is modified by increased disease chronicity.

## Introduction

Major depressive disorder (MDD) is a highly prevalent and disabling mental health condition characterized by pervasive symptoms of sadness, hopelessness, and anhedonia (American Psychiatric Association, 2013). Depression often first arises during adolescence (Gore et al., 2011; Thapar, Collishaw, Pine, & Thapar, 2012), with an early onset being a strong predictor of recurrent depression (Fergusson, Horwood, Ridder, & Beautrais, 2005; Klein, Shankman, Lewinsohn, & Seeley, 2009) as well as greater reductions in quality of life and wellbeing into adulthood (Zisook et al., 2007). Early adolescence is marked by increased use of maladaptive emotional regulation strategies which in turn are associated with exacerbated mental health issues (Cracco, Goossens, & Braet, 2017; Silk, Steinberg, & Morris, 2003). One such strategy, known as expressive suppression, which involves inhibiting the outward expression of emotional behavior (Gross, 2015), has been specifically associated with depressive symptoms severity (Larsen et al., 2013). However, much of the neurobiology of depression, particularly during this early stage of the disorder, remains unclear.

Previous neuroimaging research has attempted to uncover the neurobiological underpinnings of MDD by focusing on long-range functional connectivity, examining the temporal correlations of large-scale, spatially distinct brain networks. Within this framework, MDD has been associated with alterations across multiple intrinsic brain networks (Brakowski et al., 2017; Li et al., 2018), including the default mode (DMN), salience, and central executive networks (Dunlop, Talishinsky, & Liston, 2019; Tse et al., 2024). However, there has been increasing interest in assessing local functional connectivity profiles (Jiang & Zuo, 2016; Zuo et al., 2013), as the functional integration of local neuronal groups appears to represent a key organizing principle in the brains of higher vertebrates (Tononi, Sporns, & Edelman, 1994). Local functional connectivity measures the temporal coherence of the functional magnetic resonance imaging (fMRI) signal between a given voxel and neighboring voxels (Jiang & Zuo, 2016). Measures of this ‘localized synchrony’ provide a framework for examining functional disruptions at rest without a priori constraints, thereby allowing for data-driven identification of regional abnormalities across conditions affecting the brain (Guo et al., 2011). Moreover, regional variation in homogeneity has been associated with the hierarchical organization of information processing across the brain, and therefore, alterations to these measures may serve as key markers of human brain function (Jiang et al., 2015; Jiang & Zuo, 2016) and a sensitive tool for identifying brain alterations in mental health disorders (Canario, Chen, & Biswal, 2021; Wei et al., 2018).

Meta-analyses examining regional homogeneity in MDD have revealed inconsistent differences in the directionality of effects and regions implicated (Chen et al., 2015; Hao, Chen, Mao, Zhong, & Dai, 2019; Iwabuchi et al., 2015). Altered local connectivity has been noted across regions including the parahippocampal gyrus, insula, and medial prefrontal cortex (Chen et al., 2015; Iwabuchi et al., 2015). Other local functional connectivity measures, including functional connectivity density and dynamic regional phase synchrony, have illustrated reduced local connectivity in the anterior cingulate cortex, precuneus, hippocampus, thalamus, and insula (Ke et al., 2016; Tang et al., 2022; Zheng et al., 2022). Conversely, recent work examining first-episode MDD observed increased regional homogeneity of the hippocampus and insula but reduced local connectivity of the orbitofrontal cortex (Hao et al., 2019). It is likely that the specific method used, including how neighborhood boundaries are defined, as well as disorder chronicity, contributes to the inconsistencies observed between studies. Despite these differences, the observed alterations appear to be localized to regions implicated in affective processing and regulation (Rolls & Grabenhorst, 2008; Uddin, Nomi, Hebert-Seropian, Ghaziri, & Boucher, 2017). Of particular note is the implication of insula dysfunction across measures of local and remote functional connectivity (Iwabuchi et al., 2014; Jamieson, Harrison, Razi, & Davey, 2022; Manoliu et al., 2014; Pastrnak, Simkova, & Novak, 2021), due to its hypothesized role in the integration of autonomic, emotional, and interoceptive stimuli (Sliz & Hayley, 2012) and potential specificity in delineating MDD from bipolar disorder (Pastrnak et al., 2021).

We aimed to investigate local functional connectivity alterations in a large sample of adolescents and young adults with MDD, using the recently introduced framework – Iso-Distant Average Correlation (IDAC; Macia et al., 2018). Measures of local functional connectivity often use arbitrary and binarized neighborhood boundaries, thereby losing the capacity to describe the rich smooth spatial gradient of local fMRI correlations (Sepulcre et al., 2010; Tomasi & Volkow, 2010; Zang, Jiang, Lu, He, & Tian, 2004). IDAC assesses the average temporal correlation of one voxel with all neighboring voxels within different spatial lags, thereby overcoming these limitations (Macia et al., 2018). Based on previous findings, we hypothesize that MDD participants would illustrate increased local functional connectivity of the insula, anterior cingulate, and hippocampus and reduced local functional connectivity of the orbitofrontal cortex. Given the developmental nature of our cohort, we additionally investigated whether local connectivity was associated with measures of depressive symptom severity, and adaptive and maladaptive emotion regulation strategy use.

## Methods

One hundred and fifty-four unmedicated, help-seeking MDD participants were recruited as part of the Youth Depression Alleviation-Combined Treatment and -Augmentation trials, for full details see Davey and colleagues (2019) and Berk and colleagues (2020). MDD participants were between 15 and 25 years of age and were recruited through specialist mental health clinics in the northern and western suburbs of Melbourne, Australia. These participants had a current diagnosis of MDD, as assessed by the Structured Clinical Interview for DSM-IV Axis I Disorders (SCID; First, Spitzer, Gibbon, & Williams, 1997). Depressive symptoms were at least of a moderate level of severity, indicated by a Montgomery-Åsberg Depression Rating Scale (MADRS) score greater than or equal to 20. Exclusion criteria included a lifetime or current diagnosis of a psychotic or bipolar disorder, current treatment with antidepressant medication, or MRI contraindications including pregnancy. One hundred age and sex-matched healthy participants were also recruited through online advertisements. They had no past mental health disorder diagnoses as assessed through SCID criteria. All participants underwent the same eight-minute resting-state fMRI scan, for which they were instructed to keep their eyes closed. These scans occurred within a week of their baseline clinical assessment and prior to commencing treatment. Due to excessive head motion (three MDD participants, four controls; see below) and incidental findings (one MDD participant, one healthy control), and missing demographic data (three MDD participants, one control), a total of seven MDD participants and six controls were excluded from further analysis. This resulted in 94 healthy controls and 147 MDD participants in our final sample. This study and consent process was approved by the Melbourne Health Human Research and Ethics Committee. An informed consent form was provided to, and signed by, all participants in this study. For participants under the age of 18, both participant consent and parental consent were required.

### Image acquisition

A 3T General Electric Signa Excite system with an eight-channel phased-array head coil was used in combination with ASSET parallel imaging. The functional sequence consisted of a single shot gradient-recalled echo-planar imaging sequence in the steady state (repetition time, 2,000 ms; echo time, 35 ms; and pulse angle, 90°) in a 23 cm field-of-view, with a 64 x 64-pixel matrix and a slice thickness of 3.5 mm (no gap). Thirty-six interleaved slices were acquired parallel to the anterior-posterior commissure line with a 20° anterior tilt to better cover ventral prefrontal brain regions. The total sequence duration was 240 whole-brain echo-planar imaging volumes. The first 4 volumes from each run were automatically discarded to allow for signal equilibration. A T1-weighted high-resolution anatomical image was acquired for each participant to assist with functional time series co-registration (140 contiguous slices; repetition time, 7.9 ms; echo time, 3 ms; flip angle, 13°; in a 25.6 cm field-of-view, with a 256 x 256-pixel matrix and a slice thickness of 1 mm). To assist with noise reduction and head immobility, all participants used earplugs and had their heads supported with foam-padding inserts.

### Image preprocessing

Imaging data were transferred to a Unix-based platform that ran MATLAB Version 9.3 (The MathWorks Inc., Natick, USA) and Statistical Parametric Mapping (SPM) Version 12 v7487 (Wellcome Trust Centre for Neuroimaging, London, UK). Preprocessing followed previously reported steps for IDAC analysis (Pujol et al., 2023). In brief, for each subject functional MRI images were slice-time corrected, realigned, and co-registered to their corresponding anatomical image with an affine transformation. Spatial normalization occurred through a back-transformation process, as such the individual 3D anatomical images were segmented and registered to MNI space so that the resulting deformation fields could be applied to the IDAC maps (see below). Images were then re-sliced to 3 x 3 x 3 mm resolution and smoothed by convolving the image with a 4 x 4 x 4 mm full width at half maximum Gaussian kernel. Motion fingerprint (Wilke, 2012) was used to quantify participant head motion. Participants were excluded if movement exceeded a mean total displacement of 3 mm (~1 native voxel).

### IDAC analysis

IDAC maps describe the pattern of correlation decay propagating from each voxel across the brain. This approach differs from other methods for assessing local connectivity by using multi-distance local measures, rather than a single local measure. These multi-distance measures can uniquely detail the rich spatial structure of the cerebral cortex functional connections, as local connectivity is distance-specific to a large extent. This graded change in local functional connectivity provides a more comprehensive characterization of the brain’s functional structure and has been shown to differentiate the human cortex into regions consistent with traditional brain atlases as well as being sensitive to different brain functional states (Macia et al., 2018). For a detailed overview of IDAC, see Macia et al. (2018) and the Supplementary Methods. Whole-cortex IDAC maps were computed using the mean correlation *z*-score of each voxel with all neighboring voxels placed at increasingly spaced iso-distant intervals. IDAC maps were calculated for three iso-distant intervals, 5–10, 15–20, and 25–30 mm, and conducted separately for the right and left hemisphere in native space. These iso-distance intervals were based on the proof of concept and sensitivity analyses described in a previous paper (Macia et al., 2018). Thinner and more numerous intervals could additionally be calculated to provide a richer characterization of IDAC curves; however, these would be both noisier and more difficult to compare to the existing literature in this space (Pujol et al., 2019; Pujol et al., 2023). It is also possible to compute iso-distant intervals beyond a 30 mm radius, however, due to negative correlations also being present this may lead to cancellation effects. Covariates included in this analysis were the six rigid body realignment parameters, their first-order derivatives, average white matter, cerebrospinal fluid, and global brain signal. Motion scrubbing using the realignment parameters from preprocessing was additionally conducted to discard motion-affected image volumes (Power et al., 2014). For volumes with greater than 0.2 mm of inter-frame motion, the corresponding volume and those immediately preceding and succeeding were discarded. A discrete cosine transform filter was applied for frequencies outside the .01–0.1 Hz interval.

The resulting maps were then normalized to the Montreal Neurological Institute (MNI) space using a back-transformation process to enable group inference. RGB color overlays were used for displaying the values from the three distances simultaneously. For this study, red represented results from 5–10 mm IDAC maps, green from 15–20 mm, and blue from 25–30 mm. Overlaps of these distances are illustrated through the respective secondary colors.

### Statistical analysis

Between groups analyses were conducted using a mixed model ANOVA (group [healthy control and MDD] by distance [5–10, 15–20, and 25–30 mm]). Interaction effects were additionally modelled to examine whether the relationship between groups differed by distance, specifically between 5–10 and 15–20 mm distances or between the 15–20 and 25–30 mm distances. Following examination of the Effect of Group F contrast, post hoc t-tests were performed to examine group differences in both directions for each of the three IDAC maps. This resulted in six primary contrasts of interest. Accordingly, we adjusted the false-discovery error rate (FDR) corrected threshold to .008 (0.05/6). Thus, all results displayed were estimated with a whole-brain, FDR corrected threshold of *p* < 0.008, *k* > 10 voxels. Secondary level covariates included age, gender, and handedness.

Given the regions implicated in the between groups comparison, we then examined whether local functional connectivity changes were associated with scores from the Emotional Regulation Questionnaire (ERQ; Gross & John, 2003). The instrument assesses two methods by which people attempt to regulate their emotions: expressive suppression (“Suppression”) and cognitive reappraisal (“Reappraisal”). In addition to the aforementioned covariates, MADRS scores were included in this regression to determine whether these effects were independent of depressive symptom severity. Analyses of between-group differences for clinical and demographic characteristics were calculated using SPSS version 27 (IBMCorp., Armonk, NY). Comparisons were adjusted for multiple comparisons using the Holm-Bonferroni correction (Holm, 1979) to determine significance (*p* < 0.05).

## Results

### Group differences between demographic and clinical characteristics

Demographic and clinical characteristics of the sample are reported in Table 1. Healthy controls and MDD participants differed significantly on MADRS symptoms (*t*(233.5) = -58.80, *p* < .001), ERQ Suppression (*t*(241) = -5.94, *p* < 0.001), and Reappraisal scores (*t*(216.6) = 10.60, *p* < 0.001; see Supplementary Figure S1 for the distribution within groups). Healthy controls were shown to have an additional 1.2 years of education on average compared with MDD participants (*t*(158.22) = 4.30 *p* < 0.001).

**Table 1.** Comparison of clinical and demographic characteristics between healthy controls and major depressive disorder participants

	Healthy Controls (*N* = 94)		MDD (*N* = 147)			
Characteristics	Mean or N	*SD* or Percentage	Mean or N	*SD* or Percentage	Hedges’ g or Cramer’s V	*p*
Age (years)	20.1	2.9	19.9	2.8	.06	1.00
Education (years)	13.6	2.4	12.4	1.8	.60	<.001
Female	52	54.7	81	54.4	.01	1.00
Handedness (right)	78	82.1	139	92.7	.17	.05
Ethnicity					.23	1.00
*Caucasian*	70	73.7	122	81.9		
*Black or African*	1	1.1	1	0.1		
*Asian*	19	20.0	23	15.4		
*South American*	3	3.3	1	0.1		
*Indian*	1	1.1	1	0.1		
*Middle Eastern*	1	1.1	2	0.1		
MADRS score	2.0	2.6	32.9	5.4	–6.82	<.001
ERQ Suppression score	14.3	4.7	17.8	4.3	–.78	<.001
ERQ Reappraisal score	30.8	6.3	22.0	6.9	1.32	<.001
Age of MDD onset	–	–	15.5	2.8	–	–

### Functional structure of the cerebral cortex

**Figure 1.**
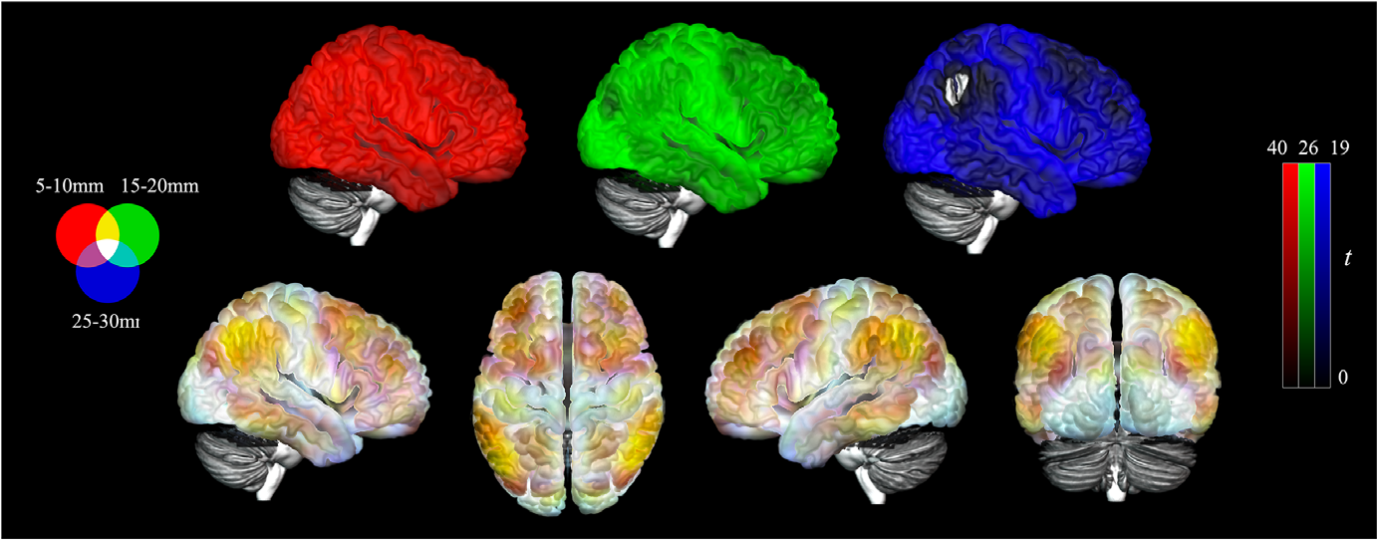
Iso-Distant Average Correlation (IDAC) brain maps across all three distances projected onto a cortical surface. Results displayed are from healthy control participants (*N* = 94) with overlay of the IDAC distances (top), 5-10 (red), 15-20 (green), and 25-30 mm (blue) with values capped at 95% of their respective maximal *t* values. The overlay of the three maps together (bottom) illustrates the consistent and unique effect across these distances through primary RGB colors and their secondary combinations. Left = Left.

To ensure that our results are consistent with previously illustrated anatomo-functional boundaries (Macia et al., 2018; Pujol et al., 2019; Pujol et al., 2023), whole-brain maps were generated across the three distances for the healthy controls (Figure 1). As observed in previous studies, the visual association cortex demonstrated high connectivity across all local distance ranges. The bilateral inferior parietal lobules, including the angular and supramarginal gyri, demonstrated high connectivity at short and medium distances. Moreover, the prefrontal cortex was composed of high connectivity in the long and medium distances.

### Group differences in the functional structure of the cerebral cortex

**Figure 2.**
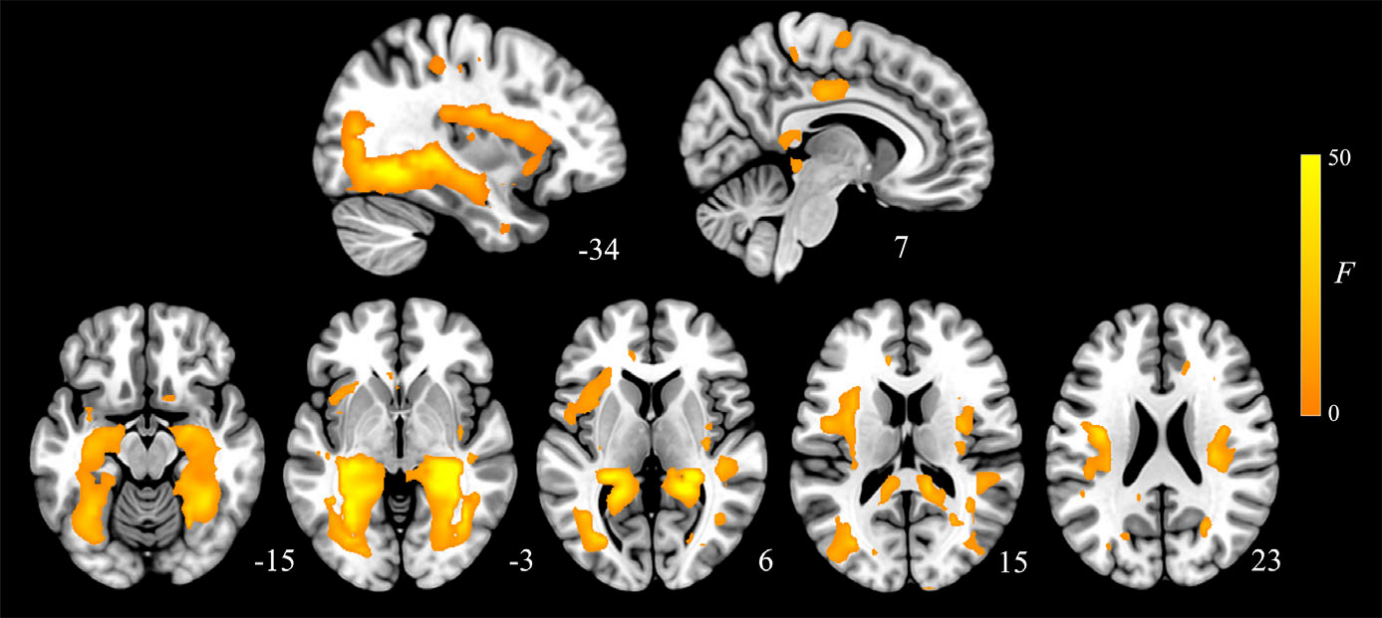
Main effects of group across the three local connectivity distances (5-10, 15-20, and 25-30 mm). Results are displayed at *p_FDR_* < 0.008, whole-brain corrected.

**Figure 3.**
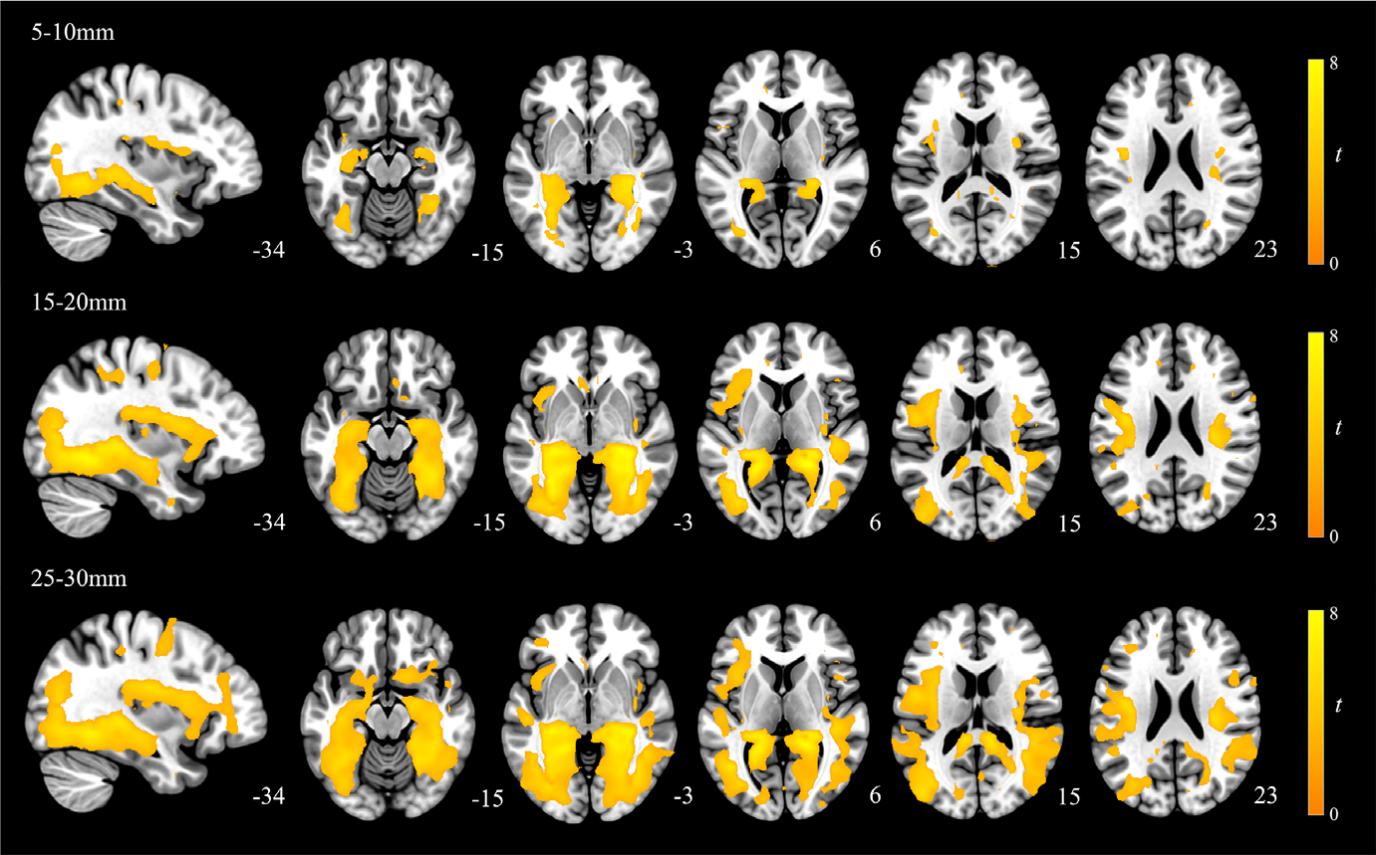
Group differences in the local functional connectivity for each of the 5–10, 15–20, and 25–30 mm distances (MDD participants > healthy controls). Results are displayed at *p_FDR_* < 0.008, whole-brain corrected.

**Figure 4.**
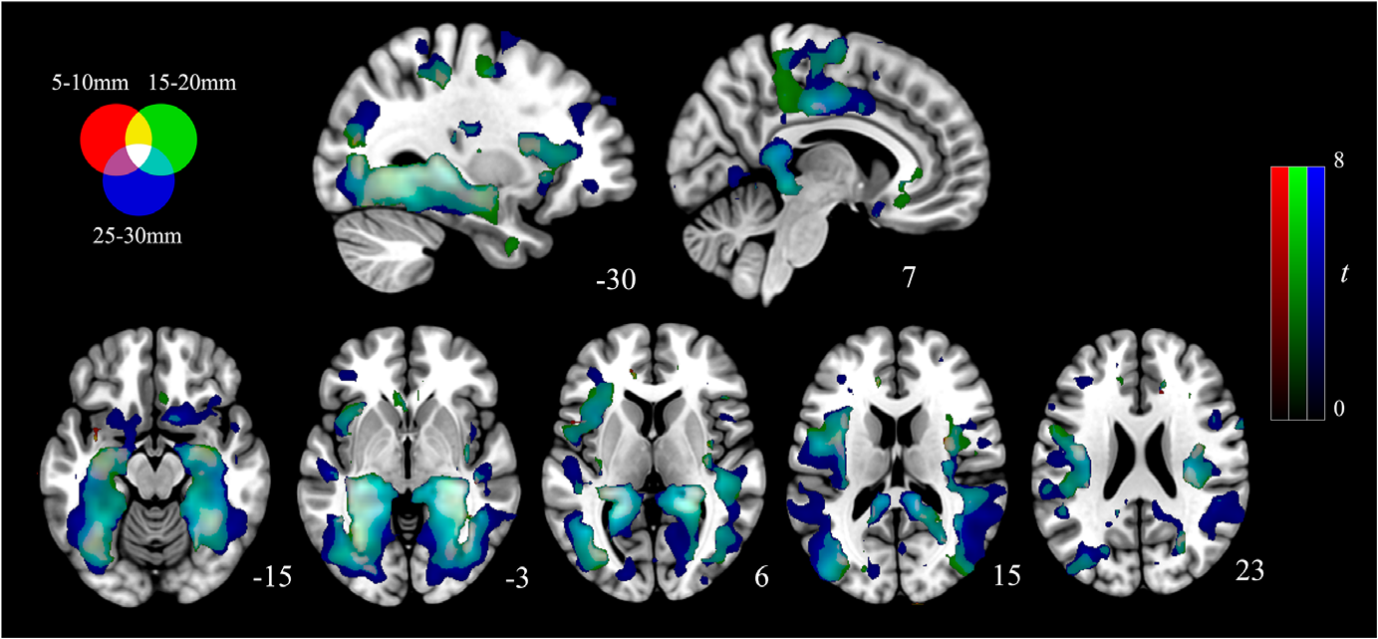
Red, green, and blue overlays illustrating the overlapping alterations to local functional connectivity present in MDD participants. Results are displayed at *p_FDR_* < 0.008, whole-brain corrected.

The Effect of Group contrast demonstrated that MDD participants and healthy controls had significant differences across the three connectivity distances (Figure 2, Supplementary Table S1). These effects were most predominately observed in the bilateral posterior hippocampus extending to the retrosplenial cortex and ventral posterior cingulate as well as to the lingual and fusiform gyri. Additional large effects were observed in the dorsal mid to posterior insular cortices and right dorsal premotor cortex. Subsequent t-test for each distance 5–10, 15–20, and 25–30 mm illustrated that these results were all driven by greater local connectivity in MDD participants compared to controls (Figure 3). As such, across all distances MDD participants demonstrated greater local functional connectivity of the bilateral extended posterior hippocampus, fusiform and lingual gyri, dorsal mid-insula, and premotor cortex (Figure 4; Supplementary Table S2, S3, and S4). We observed no significant group by distance interaction effects for either the 5–10 and 15–20 mm or the 15–20 and 25–30 mm comparison.

### The associations between emotional regulation, depressive symptoms, and local functional connectivity

We observed a significant association between ERQ suppression scores and local connectivity (Supplementary Figure S2). Specifically, there was a consistent association between these scores and the local connectivity of the left mid-insula cortex for the short and medium lengths (5–10 and 15–20 mm). MADRS scores adjusted for ERQ suppression and reappraisal scores were conversely associated with connectivity alterations in the bilateral retrosplenial cortex and hippocampi, lingual gyri, midcingulate, dorsal and ventral anterior insula, and supplementary motor cortex across all three distances (Supplementary Figure S3). No significant correlations were observed between IDAC measures and ERQ Reappraisal scores.

## Discussion

Our study observed that MDD participants had greater multi-distance local functional connectivity across several brain regions, with the largest effects observed in the retrosplenial cortex, posterior hippocampus, dorsal mid-insula, and the fusiform and lingual gyri. We did not identify reduced local connectivity in the orbitofrontal cortex as hypothesized. Additionally, we observed associations between ERQ Suppression scores and the local connectivity of the insula across participants.

### Posterior hippocampal and retrosplenial alterations

The posterior hippocampus and retrosplenial cortex have consistently shown functional alterations in depression. Meta-analyses have identified increased regional homogeneity of the left hippocampus in drug naïve MDD participants (Hao et al., 2019). Similarly, reduced suppression of the hippocampus has been observed in depressed participants during loss reinforcement learning conditions, with the degree of this hyperactivity being associated with severity of symptoms (Johnston et al., 2015). Depression has also been associated with functional alterations in the retrosplenial cortex (Harel, Tennyson, Fava, & Bar, 2016; Kumar et al., 2008; Zhu et al., 2012). Notably, both regions are components of the medial temporal subsystem of the DMN (Andrews-Hanna, Reidler, Sepulcre, Poulin, & Buckner, 2010) and have been broadly implicated in affective memory and information processing, particularly autobiographical memory (Andrews-Hanna, Smallwood, & Spreng, 2014; Miller, Vedder, Law, & Smith, 2014). The retrosplenial cortex has also been implicated in key components of episodic memory including mental imagery and projecting oneself in time (Chrastil, 2018). Thus, these regions are spatially and functionally positioned to link DMN subsystems necessary for episodic memory retrieval and core DMN regions, such as the posterior cingulate cortex, necessary for the generation of self-conceptualization (Andrews-Hanna et al., 2014; Davey & Harrison, 2022; Kaboodvand, Backman, Nyberg, & Salami, 2018; Jamieson et al., 2023). Increased local synchrony of these regions observed in depressed individuals may therefore represent changes to the processing and encoding of emotional memory (Jaworska, Yang, Knott, & MacQueen, 2015) and specifically contribute to the negatively biased recall of stimuli hypothesized by cognitive models of depression (Disner, Beevers, Haigh, & Beck, 2011).

In rodent work, the retrosplenial cortex has been implicated in abnormal long-term metabolic stress responses and vulnerability to depression (Harro et al., 2014). Excitatory efferent projections from the dorsal hippocampus, equivalent to the posterior hippocampus in humans (Fanselow & Dong, 2010), to the retrosplenial cortex have been identified as contributors to the generalization of negative memories (Ren et al., 2022). The hippocampus demonstrates a high proportion of glutamatergic pyramidal cells (Freund & Buzsaki, 1996; Olbrich & Braak, 1985), with co-activation of these neurons during rest being shown to significantly increase following chronic stress exposure (Tomar, Polygalov, Chattarji, & McHugh, 2021). Thus, due to the sensitivity of these regions to stress (Corcoran, Yamawaki, Leaderbrand, & Radulovic, 2018), hippocampus and retrosplenial cortex dysfunction may represent a potential mechanism through which early life adversity results in changes to the processing and encoding of emotional memory. This, in turn, may aid in explaining the reductions in hippocampal volume observed in older depressed participants (Huang et al., 2013; MacQueen & Frodl, 2011; Malykhin & Coupland, 2015). Future examination of how these changes in local synchrony progress as a function of the chronicity of the disorder will provide insight about the longitudinal effect these local alterations have on other regions and networks.

### Dorsal mid-insula alterations

Compared to healthy controls, MDD participants have shown decreased activity of the mid-insula cortex during interoceptive processing (Avery et al., 2014; DeVille et al., 2018) and recall (DeVille et al., 2018). It has been proposed that the insula illustrates a posterior-to-anterior progression of interoceptive processing, with the posterior insula mapping interoceptive signals (Craig, 2002; Kuehn, Mueller, Lohmann, & Schuetz-Bosbach, 2016), and the anterior insula associated with interoceptive awareness (Craig, 2009; Gu, Hof, Friston, & Fan, 2013). Within this framework, the dorsal mid-insula is well placed to serve as an intermediary between these processes. Primate cytoarchitectonics of the precentral insular gyrus further supports this functioning, as the mid-insula both projects to (Mesulam & Mufson, 1982), and receives input from (Mufson & Mesulam, 1982), the anterior and posterior insula. Moreover, the mid-insula has previously been associated with interoceptive processing (Centanni, Janes, Haggerty, Atwood, & Hopf, 2021), including both interoceptive attention (Kurth, Zilles, Fox, Laird, & Eickhoff, 2010; Schulz, 2016) and interoceptive recall (DeVille et al., 2018). Together this work indicates that this part of the insula may be responsible for processing interoceptive prediction errors, which occur following a mismatch between expectations concerning physiological states and bodily signals (Barrett & Simmons, 2015). A recent meta-analysis examining alterations across multiple psychiatric disorders during interoception identified consistent changes to the mid-insula (Nord, Lawson, & Dalgleish, 2021). As such, the integrative role of the mid-insula may increase its sensitivity to dysfunctional signals emerging from disparate bodily and brain systems (Nord et al., 2021; Sliz & Hayley, 2012), thereby contributing to abnormal emotional states (Namkung, Kim, & Sawa, 2018).

Interestingly, we observed that the local functional connectivity of the insula was associated with habitual use of expressive suppression. Due to expressive suppression involving inhibiting the outward expression of emotional behavior (Gross, 2015), its use may be viewed as an attempt to exert top-down control of interoceptive signals (Nord et al., 2021; Paulus & Stein, 2010). Maladaptive emotional regulation strategies are well noted in depressive samples (Schafer, Naumann, Holmes, Tuschen-Caffier, & Samson, 2017), including increased use of expressive suppression and decreased use of cognitive reappraisal (Joormann & Gotlib, 2010). While a recent review has highlighted mixed evidence concerning whether expressive suppression is associated with depression (Dryman & Heimberg, 2018), longitudinal research suggests that expressive suppression may both predict (Larsen et al., 2013) and be predicted by depressive symptoms (Larsen et al., 2012). The precise nature of this relationship likely depends on the developmental stage being investigated (Dryman & Heimberg, 2018). Age has been suggested as an important mediator of this relationship given that older adults use expressive suppression more frequently, however, experience less psychological distress from its use (Brummer, Stopa, & Bucks, 2014). Thus, the young nature of our sample may explain the large difference in expressive suppression observed between the healthy controls and MDD participants.

### Lack of orbitofrontal alterations

Contrary to our hypothesis we did not observe any between group differences in orbitofrontal cortex local connectivity. Previous work by Hao et al. (2019) had noted reductions in local functional connectivity of the orbitofrontal cortex in treatment-naive individuals; however, the average age of participants in this meta-analysis was 31.75 years. As such, they were on average a decade older than our participants and were likely to have been experiencing their symptoms for a longer duration. Notably, measures of local connectivity have been shown to decrease from adolescence to adulthood (Lopez-Larson, Anderson, Ferguson, & Yurgelun-Todd, 2011) and orbitofrontal regions are some of the last brain areas to mature in humans (Gogtay et al., 2004; Toga, Thompson, & Sowell, 2006). This may mean that the reductions in orbitofrontal cortex connectivity only become observable once individuals with depression are beyond this extended adolescent period and well into adulthood. Longitudinal research examining illness progression beyond this developmental period (over 25 years of age) would aid in disentangling whether this is an effect only observed in later adulthood or a function of illness duration.

### Limitations

The strength and novelty of this study should be considered in the context of its limitations.

While the focus on treatment-naïve individuals provides valuable insights into the neurobiological underpinnings of depression without the potentially confounding effects of medication, it also limits our generalizability. It remains unclear whether these same effects would be present in older populations, those with mixed medication statuses, and more variable illness chronicity. Due to the cross-sectional nature of this study, we are unable to infer whether the identified effects precede illness onset (i.e. represent risk factors for depression) or are caused by depression. Longitudinal research examining local connectivity prior to illness onset would aid in disentangling these effects. Post hoc pairwise comparisons require multiple comparisons corrections to adjust for their increased likelihood of identifying false positive effects. However, these corrections in turn increase the likelihood of false negative errors, thereby potentially impairing our ability to detect effects of interest (Garofalo, Giovagnoli, Orsoni, Starita, & Benassi, 2022). Defining these comparisons a priori, based on the experimental hypotheses, would aid in reducing the number of these comparisons and thus the false positive rate.

### Conclusion

We have examined the local functional connectivity of the cerebral cortex using a novel approach to investigate brain alterations associated with MDD. We identified differences in the local synchrony of several regions, including the retrosplenial cortex, posterior hippocampus, and dorsal mid-insula. Across these regions depressed individuals demonstrated increased coupling of intracortical activity. Additionally, across participants increased local functional connectivity of the anterior insula was associated with use of expressive suppression as an emotional regulation strategy. Longitudinal examination of these between group local synchrony differences will aid in identifying whether this relationship changes as a function of the disorder chronicity.

## Supporting information

Jamieson et al. supplementary material Jamieson et al. supplementary material

## Supplementary material

The supplementary material for this article can be found at http://doi.org/10.1017/S0033291725000510.
