# Supplementary material for: Graded changes in local functional connectivity of the cerebral cortex in young people with depression
Source: Psychol Med. 2025 Mar 17;55:e88. doi: 10.1017/S0033291725000510 (PMC12080650; doi:10.1017/S0033291725000510)
Supplement: Jamieson et al. supplementary material [file S0033291725000510sup001.docx]

**Supplementary Materials**

**Supplementary Methods……………………………………………………………………2**

**Supplementary Tables………………………………………………………………………3**

Supplementary Table S1: Group differences in Iso-Distance Average Correlation (IDAC) functional connectivity measures across the three distances (F contrast Effect of Group).….3

Supplementary Table S2: Group differences in functional connectivity measures based on Iso-Distance Average Correlations (IDAC) for the 5-10mm distance………..……………...5

Supplementary Table S3: Group differences in functional connectivity measures based on Iso-Distance Average Correlations (IDAC) for the 15-20mm distance………..…………….6

Supplementary Table S4: Group differences in functional connectivity measures based on Iso-Distance Average Correlations (IDAC) for the 25-30mm distance………..…………….8

**Supplementary Figures……………………………………………………………………10**

Supplementary Figure S1: Distribution of (A) Montgomery-Åsberg Depression Rating Scale scores, (B) Emotion Regulation Questionnaire Suppression Subscale scores and (C) Emotion Regulation Questionnaire Reappraisal Subscale scores by diagnostic group. Healthy controls are shown in blue, MDD participants in green.………..…………………..………………..10

Supplementary Figure S2: Red, green, and blue overlays illustrating associations between ERQ Suppression scores and local functional connectivity.……………………………….11

Supplementary Figure S3: Red, green, and blue overlays illustrating associations between MADRS scores and local functional connectivity.……………………………………..….12

**Supplementary Methods**


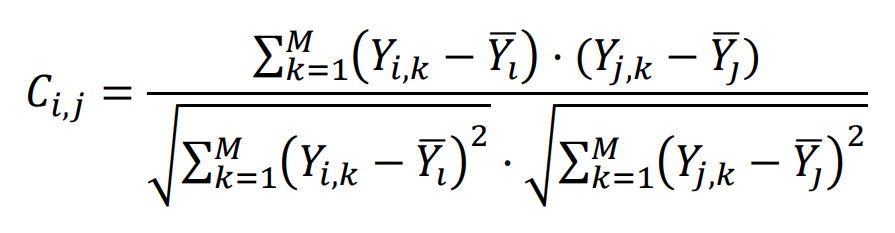
The concept of “Iso-Distant Average Correlation” (IDAC) is used to describe the pattern of correlation decay in the close vicinity of a voxel (Macia et al., 2018). IDAC_i_(*h*) was consequently defined as the average temporal correlation of voxel *i* with all the voxels located at a given Euclidean distance interval *h*. Functional MRI data sets being a discrete sample, any distance interval h must be necessarily transformed into a discrete iso-distant interval H_k_=(h_k_, h_k+1_), with h*_k_* being a set of successively increasing distances covering the whole vicinity of a given voxel. The set of iso-distant intervals H_k_ were selected so that temporal correlations were mainly positive, decreased monotonically and in which horizontal axon collaterals were considered likely to form local networks. For the present study, we defined 3 iso-distant intervals: 5-10mm, 15-20mm and 25-30mm, with constant thicknesses but increasing number of voxels. We first computed a correlation matrix *C* of Pearson coefficients comparing the functional MRI signal time course of all the voxels in our study mask with each other’s.


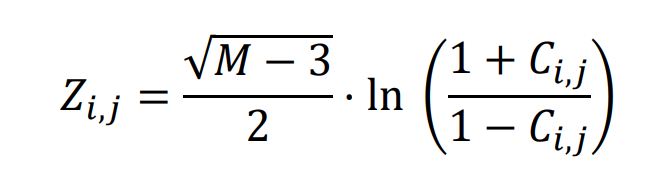
where M is the length of the functional MRI signal time series and *i* and *j* index all the voxels entering our study mask. We then transformed the Pearson correlation matrix *C* into a Gaussian distributed z-score correlation matrix Z by applying a Fisher transform.


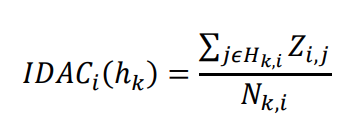
We obtained then IDAC_i_(h_k_) by averaging the correlation coefficients of voxel *i* with all the voxels *j* belonging to the interval H_k_.

In short, IDAC values are defined as the mean correlation z-score between one voxel’s functional MRI signal and the functional MRI signal of all the voxels within the iso-distant interval H_k,i_. Note that, for a given distance interval *k*, the number of voxels within the concentric iso-distant interval N_k,i_ is not necessarily the same for every voxel *i* due to the edge effects of the study mask.

| **Supplementary Tables**  Supplementary Table S1  *Group Differences in Iso-Distance Average Correlation (IDAC) Functional Connectivity Measures Across the Three Distances (F Contrast for Effect of Group)* | | | | | | | |  |
| --- | --- | --- | --- | --- | --- | --- | --- | --- |
| Brain region | Hemisphere | BA | Coordinates | | | Cluster size (3mm^3^ voxels) | *F*-value | |
|  |  |  | X | Y | Z |  |  |  |
| Hippocampus | R | - | 34 | -33 | -3 | 893 | 67.79 | |
| Retrosplenial cortex | R | 30 | 19 | -42 | 6 |  | 60.89 | |
| Retrosplenial cortex | R | 30 | 22 | -42 | -3 |  | 52.84 | |
| Fusiform gyrus | L | 37 | -35 | -54 | -9 | 964 | 61.58 | |
| Hippocampus | L | - | -29 | -30 | -3 |  | 59.58 | |
| Fusiform gyrus | L | 37 | -32 | -63 | -6 |  | 51.57 | |
| Insula | L | 13 | -35 | -9 | 21 | 151 | 45.40 | |
|  | L | 13 | -35 | 0 | 18 |  | 40.03 | |
|  | L | 13 | -32 | 9 | 12 |  | 28.72 | |
| Putamen | R | - | 31 | -6 | 12 | 68 | 43.31 | |
| Insula | R | 13 | 31 | -15 | 18 |  | 36.22 | |
|  | R | 13 | 31 | -21 | 24 |  | 35.27 | |
| Dorsal posterior cingulate cortex | L | 31 | -17 | -36 | 42 | 17 | 31.81 | |
| Frontal eye fields | L | 4 | -17 | -30 | 48 |  | 31.47 | |
| Superior temporal gyrus | R | 22 | 43 | -30 | 3 | 48 | 30.77 | |
|  | R | 22 | 43 | -39 | 18 |  | 27.63 | |
|  | R | 22 | 49 | -33 | 9 |  | 24.32 | |
| Mid cingulate cortex | R | 24 | 13 | -9 | 42 | 51 | 30.51 | |
| Supplementary motor cortex | R | 6 | 16 | -21 | 42 |  | 29.74 | |
| Ventral posterior cingulate | R | 23 | 10 | -18 | 36 |  | 27.43 | |
| Visual association cortex | R | 18 | 28 | -63 | 21 | 11 | 25.28 | |

| Supplementary Table S2  *Group Differences in Functional Connectivity Measures Based on Iso-Distance Average Correlations (IDAC) for the 5-10mm Distance* | | | | | | | |  |
| --- | --- | --- | --- | --- | --- | --- | --- | --- |
| Brain region | Hemisphere | BA | Coordinates | | | Cluster size (3mm^3^ voxels) | t-value | Hedge’s g |
|  |  |  | X | Y | Z |  |  |  |
| *MDD > Controls* |  |  |  |  |  |  |  |  |
| Hippocampus | R | - | 31 | -36 | 0 | 282 | 6.93 | 0.92 |
| Fusiform gyrus | R | 37 | 40 | -45 | -9 |  | 6.28 | 0.83 |
| Visual association cortex | R | 19 | 40 | -60 | -3 |  | 6.17 | 0.81 |
| Hippocampus | L | - | -26 | -36 | 3 | 239 | 6.44 | 0.85 |
| Fusiform gyrus | L | 37 | -35 | -60 | -9 |  | 6.43 | 0.85 |
| Hippocampus | L | - | -35 | -39 | -6 |  | 5.89 | 0.78 |
| Insula | R | 13 | 31 | -6 | 12 | 17 | 6.05 | 0.80 |
| Primary motor cortex | R | 4 | 37 | -6 | 21 |  | 5.31 | 0.70 |
| Insula | R | 13 | 34 | 3 | 18 |  | 5.18 | 0.68 |
| Insula | L | 13 | -35 | -9 | 21 | 19 | 5.63 | 0.74 |
| Pars opercularis | L | 44 | -35 | 0 | 18 |  | 5.06 | 0.67 |
| Visual association cortex | L | 19 | -29 | -75 | 0 | 23 | 5.57 | 0.74 |
|  | L | 19 | -32 | -75 | 15 |  | 5.21 | 0.69 |
|  | L | 19 | -38 | -69 | 9 |  | 5.00 | 0.66 |

| Supplementary Table S3  *Group Differences in functional connectivity measures based on Iso-Distance Average Correlations (IDAC) for the 15-20mm distance* | | | | | | | | |
| --- | --- | --- | --- | --- | --- | --- | --- | --- |
| Brain region | Hemisphere | BA | Coordinates | | | Cluster size (3mm^3^ voxels) | t-value | Hedge’s g |
|  |  |  | X | Y | Z |  |  |  |
| *MDD > Controls* |  |  |  |  |  |  |  |  |
| Hippocampus | R | - | 28 | -30 | -3 | 1187 | 8.64 | 1.14 |
| Retrosplenial cortex | R | 30 | 19 | -42 | 6 |  | 8.56 | 1.13 |
| Parahippcampal gyrus | R | 36 | 22 | -42 | -3 |  | 7.63 | 1.01 |
| Hippocampus | L | - | -29 | -30 | -3 | 1252 | 8.18 | 1.08 |
| Visual association cortex | L | 19 | -32 | -54 | -6 |  | 8.18 | 1.08 |
| Retrosplenial cortex | L | 30 | -20 | -45 | 3 |  | 7.75 | 1.02 |
| Primary motor cortex | L | 6 | -38 | -9 | 21 | 278 | 6.45 | 0.85 |
| Insula | L | 13 | -35 | 0 | 18 |  | 6.37 | 0.84 |
| Premotor cortex | L | 6 | -44 | -3 | 18 |  | 5.95 | 0.79 |
| Insula | R | 13 | 31 | -15 | 18 | 172 | 6.13 | 0.81 |
|  | R | 13 | 31 | -6 | 12 |  | 6.08 | 0.80 |
| Primary auditory cortex | R | 40 | 43 | -27 | 3 |  | 5.82 | 0.77 |
| Mid cingulate cortex | R | 24 | 13 | -9 | 42 | 106 | 5.56 | 0.73 |
| Mid cingulate cortex | R | 24 | 7 | -21 | 36 |  | 5.16 | 0.68 |
| Sensory association cortex | L | 5 | 16 | -33 | 48 |  | 4.85 | 1.14 |
| Dorsal posterior cingulate cortex | L | 31 | -17 | -30 | 48 | 35 | 5.26 | 1.13 |
| Sensory association cortex | L | 5 | -14 | -36 | 54 |  | 5.06 | 1.01 |
| Dorsal posterior cingulate cortex | L | 5 | -17 | -36 | 42 |  | 4.86 | 1.08 |
| Supplementary motor cortex | L | 6 | -14 | -12 | 45 | 13 | 5.01 | 1.08 |
| Dorsal posterior cingulate cortex | R | 5 | 28 | -63 | 24 | 11 | 4.68 | 1.02 |
| Supplementary motor cortex | R | 6 | 16 | -6 | 63 | 43 | 4.46 | 0.85 |
|  | R | 6 | 1 | -12 | 66 |  | 4.36 | 0.84 |
|  | R | 6 | 22 | -12 | 60 |  | 4.30 | 0.79 |

| Supplementary Table S4  *Group Differences in Functional Connectivity Measures Based on Iso-Distance Average Correlations (IDAC) for the 25-30mm Distance* | | | | | | | | |
| --- | --- | --- | --- | --- | --- | --- | --- | --- |
| Brain region | Hemisphere | BA | Coordinates | | | Cluster size (3mm^3^ voxels) | t-value | Hedge’s g |
|  |  |  | X | Y | Z |  |  |  |
| *MDD > Controls* |  |  |  |  |  |  |  |  |
| Retrosplenial cortex | R | 30 | 19 | -42 | 6 | 1787 | 8.10 | 1.07 |
|  | R | 30 | 34 | -33 | -3 |  | 7.58 | 1.00 |
| Hippocampus | R | - | 22 | -33 | 0 |  | 7.40 | 0.98 |
| Fusiform gyrus | L | 37 | -38 | -51 | -9 | 1583 | 7.74 | 1.02 |
| Hippocampus | L | - | -29 | -30 | -3 |  | 7.73 | 1.02 |
| Retrosplenial cortex | L | 3 | -20 | -45 | 0 |  | 7.67 | 1.01 |
| Primary motor cortex | L | 4 | -35 | -9 | 21 | 299 | 6.49 | 0.86 |
| Premotor cortex | L | 5 | -47 | -3 | 18 |  | 5.96 | 0.79 |
| Insula | L | 13 | -35 | 0 | 18 |  | 5.83 | 0.77 |
| Ventral posterior cingulate cortex | R | 23 | 10 | -18 | 36 | 188 | 5.90 | 0.78 |
| Mid cingulate cortex | R | 24 | 16 | -21 | 42 |  | 5.61 | 0.74 |
|  | L | 24 | -14 | -12 | 45 |  | 5.20 | 0.69 |
| Supplementary motor area | R | 6 | 34 | -3 | 54 | 106 | 5.27 | 0.70 |
|  | R | 6 | 34 | -6 | 45 |  | 5.02 | 0.66 |
|  | R | 6 | 43 | 3 | 33 |  | 4.17 | 0.55 |
| Primary auditory cortex | L | 41 | -47 | -24 | 0 | 26 | 5.14 | 0.68 |
| Supplementary motor cortex | L | - | -20 | -6 | 69 | 41 | 4.91 | 0.65 |
| Primary sensory cortex | R | 1 | 55 | -18 | 54 | 16 | 4.75 | 0.63 |
| Subgenual cingulate cortex | R | 25 | 10 | 12 | -18 | 38 | 4.74 | 0.63 |
| Putamen | R | - | 25 | 12 | -12 |  | 4.54 | 0.60 |
| Insula | R | 13 | 37 | 12 | -12 |  | 4.33 | 0.57 |
| Supplementary motor cortex | R | 6 | 16 | -3 | 66 | 24 | 4.71 | 0.62 |
| Dorsal posterior cingulate cortex | R | 31 | 25 | -63 | 27 | 15 | 4.55 | 0.60 |
| Mid cingulate cortex | L | 24 | -8 | 6 | 33 | 23 | 4.44 | 0.59 |
|  | L | 24 | -2 | 0 | 42 |  | 3.96 | 0.52 |

Supplementary Figures


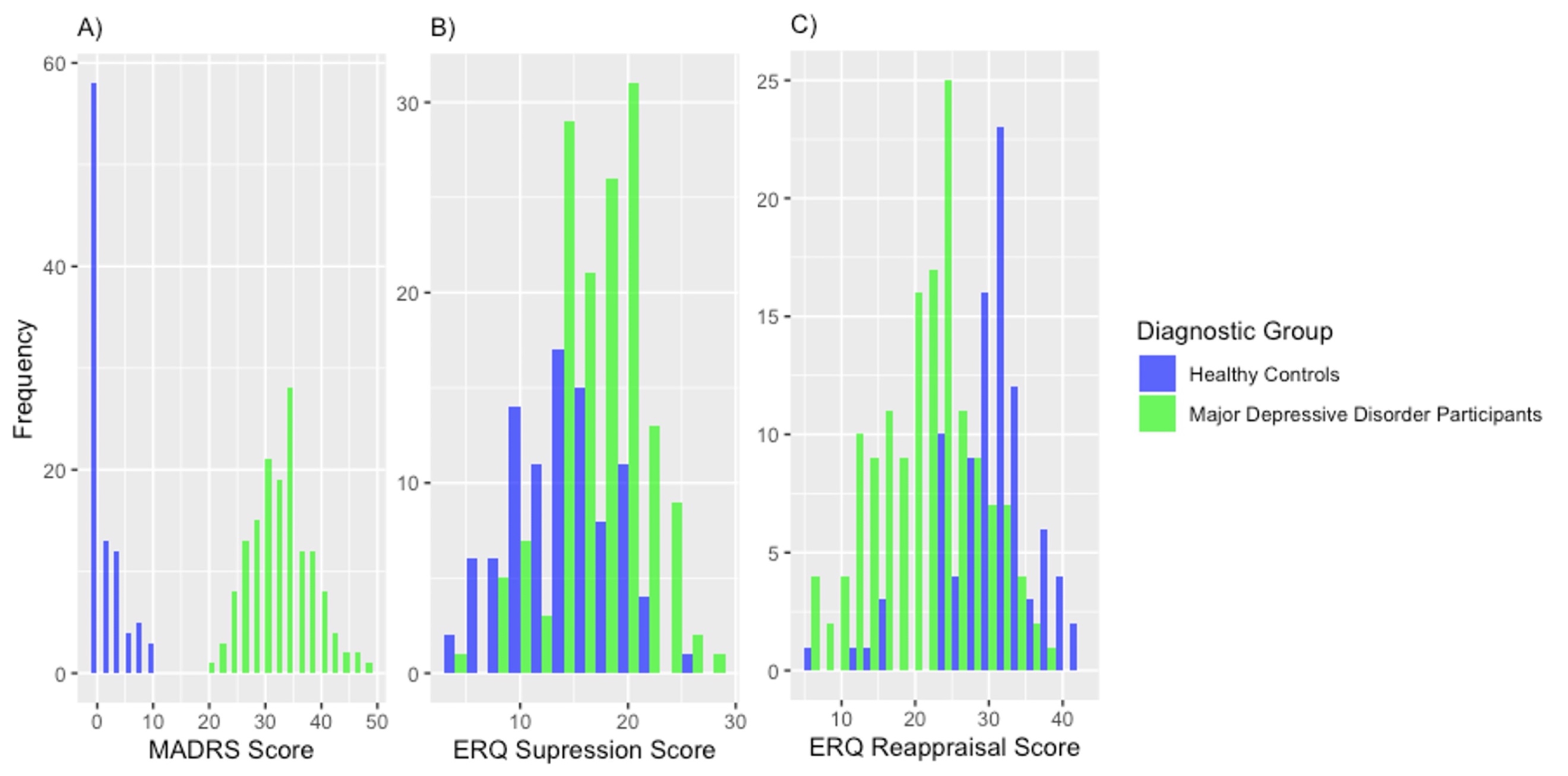


*Supplementary Figure S1.* Distribution of (A) Montgomery-Åsberg Depression Rating Scale scores, (B) Emotion Regulation Questionnaire Suppression Subscale scores and (C) Emotion Regulation Questionnaire Reappraisal Subscale scores by diagnostic group. Healthy controls are shown in blue, MDD participants in green.

**
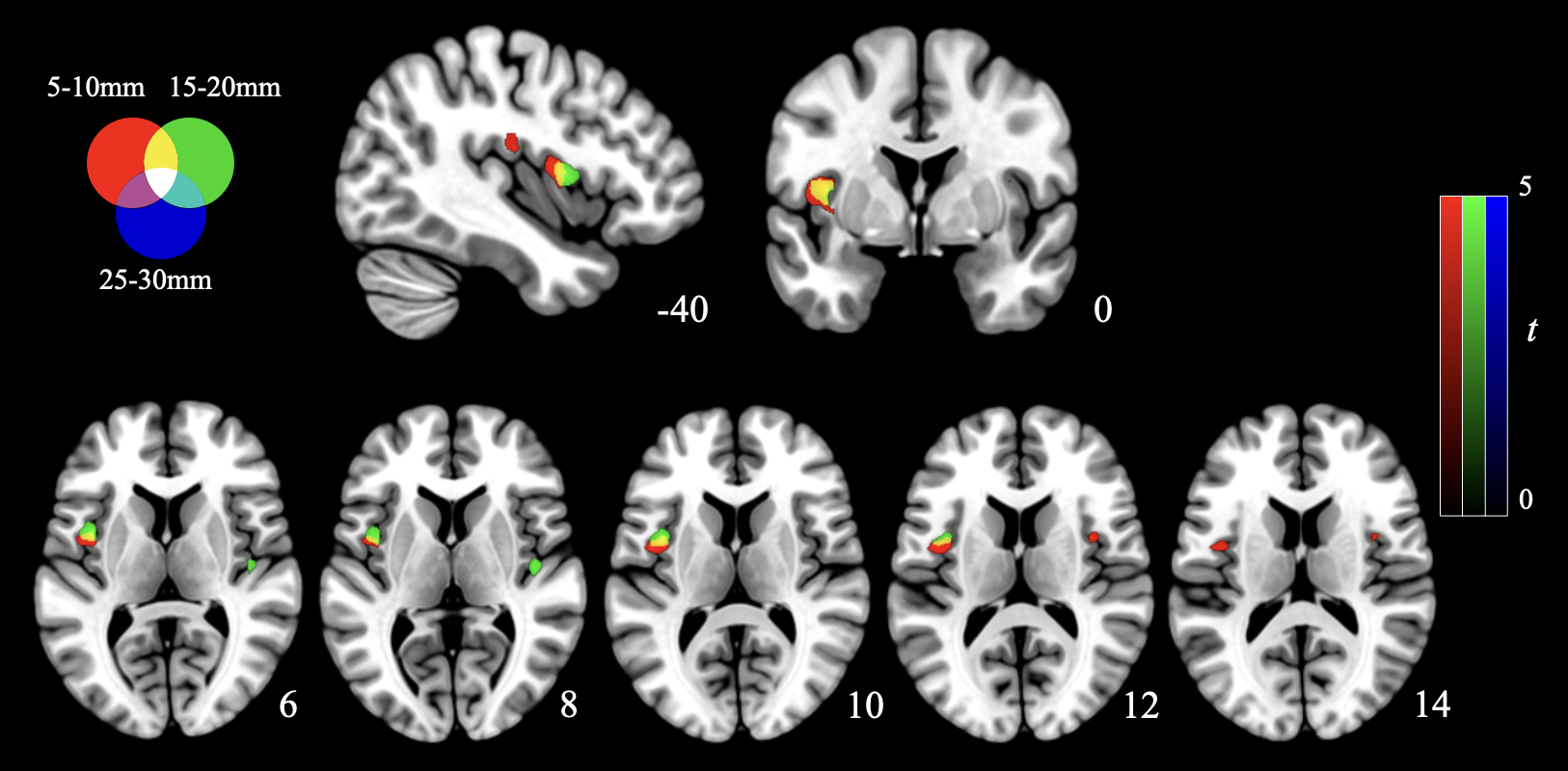
**

*Supplementary Figure S2.* Red, green, and blue overlays illustrating associations between ERQ Suppression scores and local functional connectivity. Results are displayed at *p* < .001, family-wise error cluster corrected.


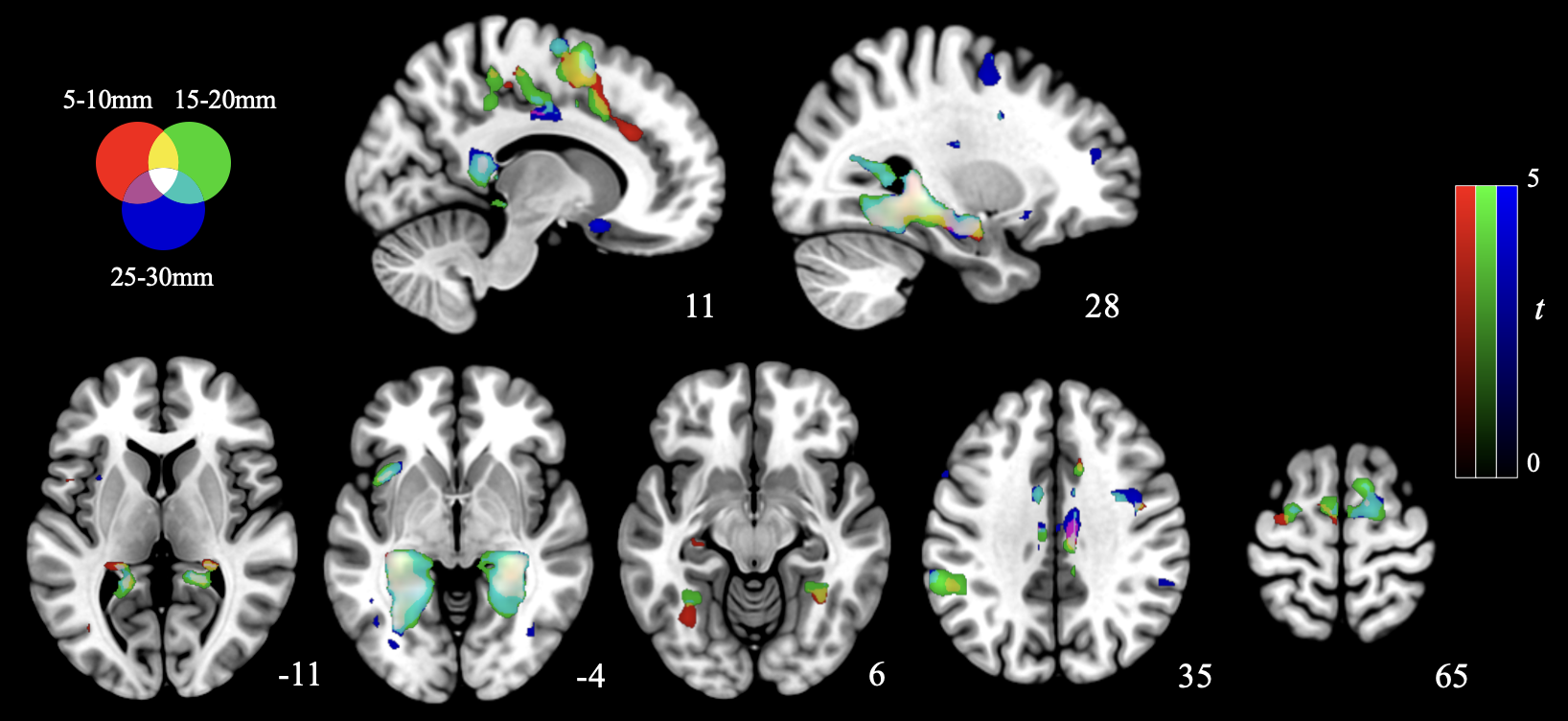


*Supplementary Figure S3.* Red, green, and blue overlays illustrating associations between MADRS scores and local functional connectivity. Results are displayed at *p* < .001, family-wise error cluster corrected.

**Supplementary References**

Macia, D., Pujol, J., Blanco-Hinojo, L., Martinez-Vilavella, G., Martin-Santos, R., & Deus, J. (2018). Characterization of the Spatial Structure of Local Functional Connectivity Using Multidistance Average Correlation Measures. *Brain Connect, 8*(5), 276-287. doi:10.1089/brain.2017.0575
